# In Situ Visualization of Localized Surface Plasmon Resonance‐Driven Hot Hole Flux

**DOI:** 10.1002/advs.202001148

**Published:** 2020-08-06

**Authors:** Hyunhwa Lee, Kyoungjae Song, Moonsang Lee, Jeong Young Park

**Affiliations:** ^1^ Department of Chemistry Korea Advanced Institute of Science and Technology (KAIST) Daejeon 34133 Republic of Korea; ^2^ Center for Nanomaterials and Chemical Reactions Institute for Basic Science (IBS) Daejeon 31414 Republic of Korea; ^3^ Research Center for Materials Analysis Korea Basic Science Institute (KBSI) Daejeon 34133 Republic of Korea

**Keywords:** finite‐difference time‐domain simulations, hot holes, localized surface plasmon resonance, photoconductive atomic force microscopy, photocurrents

## Abstract

Nonradiative surface plasmon decay produces highly energetic electron–hole pairs with desirable characteristics, but the measurement and harvesting of nonequilibrium hot holes remain challenging due to ultrashort lifetime and diffusion length. Here, the direct observation of LSPR‐driven hot holes created in a Au nanoprism/p‐GaN platform using photoconductive atomic force microscopy (pc‐AFM) is demonstrated. Significant enhancement of photocurrent in the plasmonic platforms under light irradiation is revealed, providing direct evidence of plasmonic hot hole generation. Experimental and numerical analysis verify that a confined |*E*|‐field surrounding a single Au nanoprism spurs resonant coupling between localized surface plasmon resonance (LSPR) and surface charges, thus boosting hot hole generation. Furthermore, geometrical and size dependence on the extraction of LSPR‐driven hot holes suggests an optimized pathway for their efficient utilization. The direct visualization of hot hole flow at the nanoscale provides significant opportunities for harnessing the underlying nature and potential of plasmonic hot holes.

## Graphical abstract

This work demonstrates that fast‐disappearing hot holes are directly visualized by utilizing a Au nanoprism/p‐GaN platform. Experimental and numerical analysis show that LSPR‐field enhancement boosts hot hole generation at the Au edge. In situ observation of hot hole flux exhibits LSPR‐hot hole interactions to serve as a starting point for the feasibility of hot hole‐based optoelectronic applications.

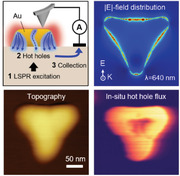

## 1 Introduction

Hot carriers are in a highly excited state and out of thermal equilibrium while confined to a metal surface during photon absorption and exothermal chemical reactions.^[^ ^1^, ^2^, ^3^, ^4^, ^5^, ^6^, ^7^ ^]^ Since carriers with high‐kinetic energies of 1−3 eV away from the Fermi energy of the metal relax to phonon scattering within tens of femtoseconds,^[^ ^2^, ^3^, ^4^, ^8^, ^9^, ^10^, ^11^, ^12^ ^]^ and disappear via energy dissipation into the surrounding materials, it has been difficult to deal with the fast‐disappearing charges from the metal surface. However, recent research has proposed that these hot carriers could be applied to effective energy converters in optoelectronic and photocatalytic applications.^[^ ^1^, ^3^, ^4^, ^6^, ^12^, ^13^, ^14^, ^15^, ^16^, ^17^, ^18^, ^19^, ^20^, ^21^, ^22^, ^23^, ^24^, ^25^, ^26^, ^27^ ^]^ To promote the generation of hot carriers with applications in photovoltaics, photodetection, and photoelectrochemical devices, pioneering studies demonstrated the key role that localized surface plasmon resonance (LSPR) plays in hot‐electron‐based energy conversion.^[^ ^6^, ^9^, ^12^, ^28^, ^29^, ^30^, ^31^, ^32^ ^]^ It has been established that the LSPR‐driven hot carriers expand the boundary of performance of efficient near‐infrared (NIR) detection of silicon‐based photodiodes,^[^ ^21^ ^]^ improve photoconversion efficiency,^[^ ^20^, ^33^, ^34^ ^]^ and enhance selectivity for CO production over hydrogen evolution.^[^ ^14^ ^]^

Compared to hot electrons, photogenerated hot holes have more energetic kinetics, a feature attributed to the asymmetrical energy distribution between hot electrons and holes relative to Fermi level,^[^ ^14^, ^29^ ^]^ and they are more suitable for efficient energy conversion. However, the short lifetime (*t* <10 ps) and mean‐free path (*l*_mfp_ ≈ 5–10 nm) of hot holes compared to hot electrons impose an obstacle to harnessing their positive characteristics.^[^ ^10^, ^14^ ^]^ As a result, relatively few studies on hot holes from plasmonic metal have been conducted either by theoretical simulations or by experimentally measuring the averaged hot hole flux in the target area,^[^ ^14^, ^35^, ^36^, ^37^, ^38^, ^39^ ^]^ despite the marvelous potential for next‐generation applications; this is in sharp contrast to the research on hot electron collection and conversion.^[^ ^2^, ^4^, ^5^, ^6^, ^12^, ^15^, ^16^, ^17^, ^22^, ^23^, ^25^, ^26^, ^30^, ^32^, ^34^, ^40^ ^]^ Furthermore, local observation of plasmonic hot holes at the nanoscale has not been demonstrated, in spite of its importance of fundamental understanding of hot carrier physics.

Here, we demonstrate in situ visualization of hot holes driven by surface plasmon at the nanoscale. By measuring the photocurrent on a Au nanoprism/p‐GaN Schottky nanodiode structure with pc‐AFM, LSPR‐driven hot holes are effectively captured and visualized. The fingerprints of the hot holes render a promise for use in ultrahigh sensitive photodetectors. We believe that this study will offer a building block for the productive application of LSPR‐coupled hot holes.

## 2 Results

### 2.1 Optical and Electrical Characterization of Plasmonic Au/p‐GaN Schottky Nanodiode

**Figure 1:**
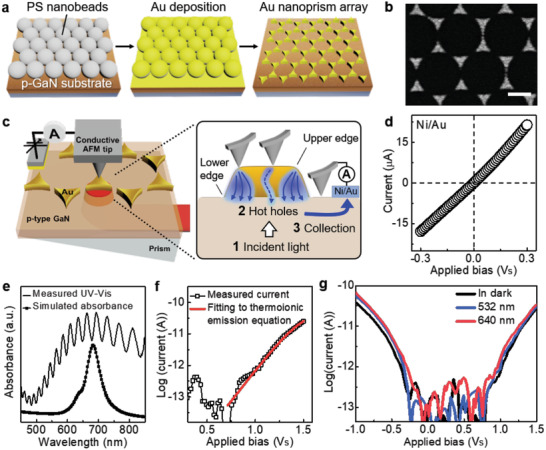
AFM measurement to collect hot holes from single Au nanoprism on p‐type GaN substrate. a) Sample fabrication steps to form the hexagonal close‐packed array of Au nanoprisms on p‐GaN substrate using self‐assembly based nanosphere lithography technique. A monolayer of polystyrene (PS) nanobeads with a diameter of 460 nm was formed on p‐GaN substrate. After ultrasonic treatment to remove gold‐coated PS nanobeads, the Au nanoprism array was fabricated. b) SEM image showing the hexagonal close‐packed Au array on p‐GaN (scale bar, 250 nm). c) Schematic illustrating photoconductive AFM to collect hot hole flux from single Au nanoprism. Generated hot holes transfer gold to p‐GaN, overcoming Schottky barrier between gold and p‐GaN. After then, hot hole flux is converted as photocurrents, which is collected by the electrical circuit in AFM system. d) *I*–*V* curve of the ohmic contact on the p‐GaN substrate formed by deposition of an Ni/Au alloy. e) Absorbance spectra of the Au nanoprism array showing localized surface plasmon resonance near 680 nm, corresponding to calculated absorbance by FDTD simulations. f) *I*–*V* curve of single Au nanoprism/p‐GaN in AFM system revealing the Schottky barrier height of 1.03 eV by fitting to thermionic emission equation. g) *I*–*V* curves at the edge of Au nanoprism/p‐GaN under 640 and 532 nm illumination with intensity of 1000 mW cm^−2^.

We constructed plasmonic Au nanoprism/p‐GaN structures to evolve LSPR‐driven hot holes (**Figure** **1**a). To collect hot holes from the plasmonic platforms, we chose p‐type c‐plane gallium nitride (GaN) as a support material below metal nanostructures. P‐type GaN materials are suitable for extracting hot holes, because they exhibit p‐type conductivity and controllable p‐type dopant concentration. Further, a Schottky barrier height over 1 eV across the Au/p‐GaN interface enables the selection of more energetic hot holes deep below the metal Fermi level.^[^ ^10^, ^29^, ^41^, ^42^ ^]^ Fabrication details for the plasmonic templates is given in the Experimental Section. The triangular Au nanoprisms on the p‐GaN/c‐plane sapphire substrate were formed by nanosphere lithography with polystyrene nanobeads (*d* ≈ 460 nm).^[^ ^43^ ^]^ These Au nanoprisms were arranged as a hexagonal close‐packed array (Figure 1b) with a thickness of 20 nm and an average side length of 136 nm (Figure S1, Supporting Information). The photocurrent was collected through the PrIr‐coated AFM tip combined with back‐illuminated lasers at wavelengths of 532 and 640 nm (OBIS series, Coherent) (Figure 1c). This configuration of Au nanoprism on p‐GaN formed a complete plasmonic Schottky nanodiode that functioned as a platform to obtain direct evidence of hot holes driven by LSPR excitation. The plasmonic hot holes would be transferred from Au to p‐GaN support over the Schottky barrier at the interface and collected through the diodes. Once hot holes are generated on the Au nanoprism, because of the short mean free path, they are transported through the Schottky barrier between Au and p‐type GaN, while low energy hot holes that fail to surmount the barrier are back supplied from the Pt/Ir AFM tip that forms Ohmic contact with the Au nanoprism, and this scheme completes the overall circuit. The charge transport in the platform occurs near the proximity of AFM tip that gives rise to the nanoscale spatial resolution of mapping the photocurrent. Current–voltage (*I*–*V*) characteristics clearly verify that ohmic contact of the device was formed by Ni/Au alloy (Figure 1d).^[^ ^44^ ^]^ To confirm the LSPR peak of the Au nanoprism/p‐GaN architecture, we measured the UV–vis absorbance spectra of the platform (Figure 1e, black solid line). The peak position was centered at a wavelength of 680 nm, accompanied by a broad curve that originated from different orientations of metallic Au arrays. The peak of numerical calculations for absorbance for a uniformly formed array of plasmonic Au nanoprisms matches the experimentally measured figure at around 680 nm, implying that the plasmonic nanostructure is adequately constructed (Figure 1e, black dot line). The Schottky barrier height at the interface between the Au nanoprism and p‐GaN support and ideality factor at room temperature are established at 1.03 and 2.61 by fitting the *I*–*V* curve to the thermionic emission equation, respectively (Figure 1f).^[^ ^45^, ^46^ ^]^ This indicates that the diode is well constructed. Note that the barrier height is close to the ideal, 1.2 eV.^[^ ^41^ ^]^ Detailed fitting parameters are in Table S1 (Supporting Information). We also employed Kelvin probe force microscope (KPFM) measurement to elucidate the charge distribution on the surface of the metal and p‐type semiconductors; it shows a potential difference of 0.9 V between Au and p‐GaN, confirming apparent metal/p‐type semiconductor junctions (Figure S2, Supporting Information).^[^ ^40^, ^47^ ^]^ Figure 1g shows the *I*–*V* characteristics collected from the edge of a Au nanoprism at the dark condition (black), 532 nm (blue), and 640 nm (red) laser illumination with a power intensity of 1000 mW cm^−2^. It is notable that the configuration illuminated with a wavelength of 640 nm in both forward and reverse voltages exhibited the highest current values in the entire measurement range, indicating that the platform strongly exhibits LSPR at a wavelength of 640 nm. The forward bias curves clearly show that the majority carriers from p‐GaN to Au govern the current transport. Small increments of photocurrent with increasing forward bias indicate that the LSPR‐driven hot holes take part in charge flux. In the reverse‐biased voltage regime, the hot hole flux increased even further beyond the dark current by the increment of the reverse bias. This implies that more hot holes move smoothly from Au to p‐GaN due to Schottky barrier height lowering (see Figure S3, Supporting Information).^[^ ^46^, ^48^ ^]^ Note that barrier height governs the probability for hot holes to be extracted as photocurrents through the LSPR‐induced platforms.

### 2.2 Direct Visualization of LSPR‐Induced Hot Hole Flux

**Figure 2:**
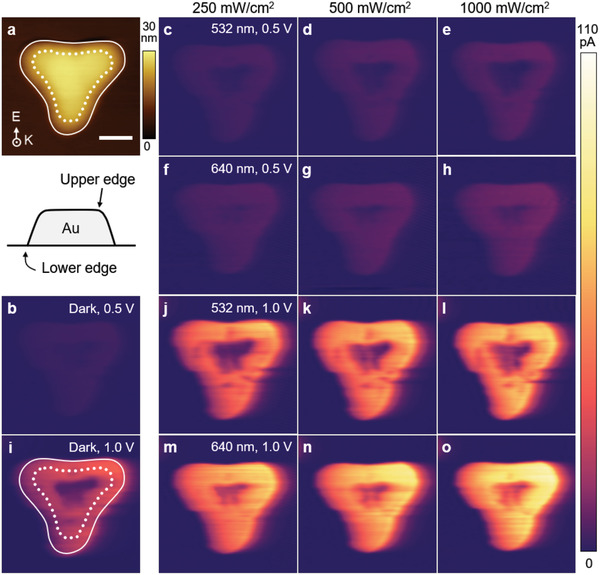
Direct observation of surface plasmon induced hot hole flux on Au nanoprism/p‐GaN nanodiode under reverse bias. a) Topography of a 136 nm long‐Au nanoprism with a thickness of 20 nm (Scale bar, 50 nm). Note a white dashed line and a white solid line represent the trace of upper edges, and that of the lower edge of a Au nanoprism, respectively; the inset shows the exact position of these edges. b) Dark current mapping under a reverse bias of 0.5 *V*_tip_ to compare photocurrent mappings. c–h) Photocurrent mappings under 0.5 *V*_tip_ with intensities of c,f) 250 mW cm^−2^, d,g) 500 mW cm^−2^, and e,h) 1000 mW cm^−2^ at 532 and 640 nm laser, respectively, exhibiting that hot holes at the upper edge were slightly increased under light illumination at 640 nm in (f)–(h). i) Current mapping in the dark condition under 1.0 *V*_tip_. j–o) Photocurrent mappings under 1.0 *V*_tip_ with intensities of j,m) 250 mW cm^−2^, k,n) 500 mW m^−2^, and l,o) 1000 mW cm^−2^ at 532 and 640 nm laser, respectively, showing that photoinduced hot holes at the upper edge were enhanced under LSPR excitation at a wavelength of 640 nm.

To shed light on the nature of LSPR‐driven hot holes, we visualized the current mapping inherited from LSPR‐induced hot holes at different incident laser wavelengths (**Figure** **2**). Current mappings were measured at different intensities of 250, 500, and 1000 mW cm^−2^ in incident laser wavelengths of the dark, 532 nm, and 640 nm illumination. The effective normal‐load (0.02 nN) of a PtIr coated probe (PPP‐CONTPt, Nanosensors) in the pc‐AFM instrument was used. The AFM image in Figure 2a showed the morphological information of an Au nanotriangle with a side length of 136 nm. Here, a white dashed line and a white solid line represent the trace of upper edges and that of the lower edge of a Au nanoprism, respectively, and the inset shows the exact position of these edges. Also, it is clearly observable that there were no distinguishable photocurrents, indicating that the hot hole transport from Au to p‐GaN is not efficient, presumably associated with the short mean free path of holes,^[^ ^29^, ^49^ ^]^ and defects localized at the interface between a Au and a p‐GaN substrate (Figure S4, Supporting Information). To extract LSPR‐driven hot holes through the Au/p‐GaN nanodiode, we applied the reverse bias of Au/p‐GaN nanodiodes to an AFM tip to reduce the Schottky barrier height at the interface. Dark current under a reverse bias of 0.5 V was ascribed to the existence of leakage currents surrounding the Au (Figure 2b). Compared to the dark current, photocurrents at 532 nm (Figure 2c–e) and 640 nm light (Figure 2f–h) under a reverse bias of 0.5 V rose only negligibly. Meanwhile, under a reverse bias of 1.0 V, photocurrents were more prominent than the dark currents (Figure 2i). The hot hole flux on 532 nm (Figure 2j–l) and 640 nm excitation (Figure 2m–o) gradually increased with the intensity of light. It is notable that the hot hole flux pumped with a wavelength of 640 nm exhibited higher current values than that of 532 nm, corresponding to UV‐Vis absorbance measurement with a maximum resonant peak position of 680 nm. This confirms that the current signal measured on the plasmonic nanostructure reflected hot hole flux driven by LSPR.

### 2.3 Boosting Hot Hole Generation at the Edge by LSPR Confinement

**Figure 3:**
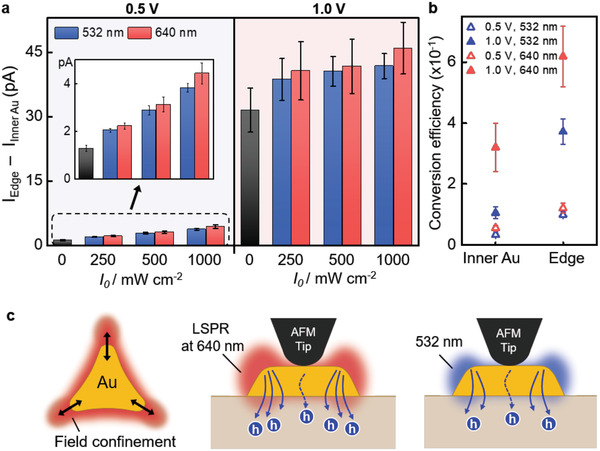
Energy conversion from incident light to hot holes on Au nanoprism/p‐GaN nanodiode. a) The difference between currents at the top edge and the inner Au under reverse bias in Figure 2. Under reverse bias, 0.5 *V*_tip_, and 1.0 *V*_tip_, the energy band bending between gold and p‐GaN are adjusted by Schottky barrier lowering, which promotes the collection of hot holes generated by LSPR excitation at 640 nm. b) The conversion efficiency of measured photocurrents under reverse bias depending on the position at inner and upper edge site. It shows the increase of conversion efficiency at the edge upon 640 nm excitation. c) Illustration of electromagnetic field distribution at the edge suggesting that generation of hot carriers is enhanced by the LSPR field‐confinement at 640 than 532 nm.

To determine the energy conversion for hot hole generation, we estimated hot hole flux for both the inner and edge sites under applied reverse voltages of 0.5 and 1.0 V with a power density of 1000 mW cm^−2^. The average photocurrent values, excluding dark currents at the edge, for wavelengths of 532 and 640 nm under the reverse bias of 0.5 and 1.0 V were 3.7, 13.8, 5.4, and 27.7 pA, respectively. Meanwhile, the average photocurrent values at the inner site under corresponding conditions exhibited 1.1, 3.6, 2.3, and 13.3 pA, respectively. Therefore, the difference of hot hole flux between the inner and the edge upon 532 and 640 nm light are 10.2 and 14.4 pA, respectively, under the bias of 1 V (see Table S2, Supporting Information). The overall values of induced photocurrents from low energetic photons with a wavelength of 640 nm had a greater difference between the current values at the edge and those inside, compared to photons with a wavelength 532 nm; the photocurrent‐difference between the inner site and the edge on 640 nm light was 1.10 and 1.06 times higher than on 532 nm light under a reverse bias of 0.5 and 1.0 V, respectively (**Figure** **3**a). This implies that the generation of plasmonic hot holes at the edge is significantly accelerated, since field‐confinement surrounding a Au nanoprism leads to the amplification of LSPR‐coupled hot hole generation. Thus, we suggest that LSPR field‐confinement at the border of a Au nanoprism can promote light absorption by increasing hot hole generation. In order to clarify the quantification of hot hole flux as a function of the contact position, photon to hot hole conversion efficiency under applied reverse bias was calculated by the following^[^ ^12^, ^50^ ^]^

(1) $$\begin{array}{ccc} \mathrm{Conversion}\;\mathrm{efficiency} & = & \frac{\#\;\mathrm{of}\;\mathrm{collected}\;\;\mathrm{electrons}\;\mathrm{per}\;\mathrm{second}}{\#\;\mathrm{of}\;\mathrm{incident}\;\mathrm{photons}\;\mathrm{per}\;\mathrm{second}}\\ & = & \frac{\mathrm{Current}\times 6.22\;\times 10^{18}}{\mathrm{Total}\;\mathrm{energy}/\mathrm{Energy}\;\mathrm{of}\;1\;\mathrm{photon}} \end{array}$$

where the total energy is 8.01 × 10^−11^ J s^−1^, currents are obtained by subtracting the dark current from the photocurrent value, and energies per photon are 3.73 × 10^−19^ and 3.10 × 10^−19^ J at 532 and 640 nm, respectively (Figure 3b). Although the conversion efficiency was calculated by considering that the contact area between a tip and the edge was 1.08 times larger than the inner Au (see Table S3, Supporting Information), the efficiency at the edge was on average 2.66 times higher than the inner site. This indicates that more hot holes are extracted from the edge of Au nanoprisms than from inside. In other words, current boosting at the edge is not promoted by the measurement area, but by confined‐surface plasmon surrounding the border of the Au nanoprism. A localized coupling between Au nanostructure and surface plasmon resonance leads to improvement of light absorption at the edge compared to the state under nonresonant light illumination (Figure 3c). This suggests that LSPR‐driven hot holes can be effectively squeezed over the potential barrier with the geometrical guidance of plasmonic structure.

### 2.4 Theoretical Prediction by FDTD Simulations for a Single Au Nanoprism

**Figure 4:**
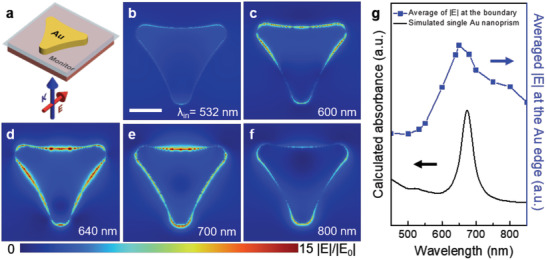
Spatial distribution of |*E*| field surrounding Au nanoprism on p‐GaN calculated by FDTD simulations. a) Schematic illustrating calculation conditions with linearly polarized light parallel to the centerline of the Au structure. |*E*| distributions for Au nanoprism under b) 532 nm, c) 600 nm, d) 640 nm, e) 700 nm, and f) 800 nm light illumination, respectively. Corresponding to absorbance spectra (Figure 1e) and current mappings (Figure 2), the amplification of *E*‐field at the edge is remarkable upon 640 nm illumination. g) Average value of *E*‐field at the edge in (b–f) with the maximum value at 650 nm (blue dot line) in accordance with theoretical calculations of the absorbance of single Au nanoprism (black solid line), revealing that LSPR excitation causes field amplification at the edge. Scale bar, 50 nm.

To resolve the origin of hot hole amplification induced by LSPR field confinement, we visualized theoretical *E*‐field distribution by utilizing finite‐difference time‐domain (FDTD) simulations (FDTD solutions, Lumerical Solutions) for a single Au nanoprism with a side length of 136 nm. Spatial |*E*| distributions were performed with linearly polarized light parallel to the centerline of Au nanoprism (**Figure** **4**a). The simulations were calculated under light excitation at wavelengths from 532 to 800 nm (Figure 4b–f). The *E*‐field distribution at the edge increased gradually by the increment of incident photon wavelengths and decreased after the peak point at 640 nm. It is essential to note that the spatial distributions of *E*‐field at the wavelength of 640 nm exhibited maximized signals, indicating that a resonant coupling between surface plasmon and field‐confinement by the geometric shape occurs with strong light absorption under incident light with a wavelength of 640 nm. This corresponds to the calculated absorbance of a single Au nanoprism with a maximum peak near 670 nm (Figure 4g, black solid line). Also, average *E*‐field value at the edge from |*E*| distributions (Figure 4g, blue dot line) showed that *E*‐field amplification maximized at a wavelength of 650 nm, revealing that field‐confinement under LSPR excitation leads directly to a remarkable increase in hot hole flux.

### 2.5 Geometrical Dependence of LSPR‐Driven Hot Hole Flux

**Figure 5:**
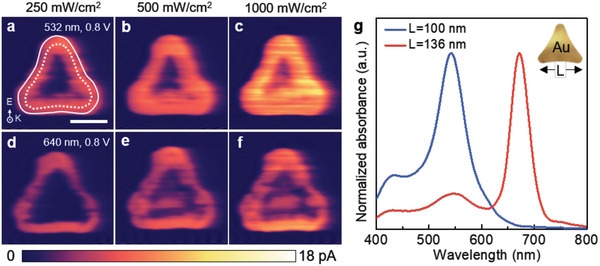
Comparative experiment of LSPR‐induced hot hole flux on small Au nanoprism/p‐GaN nanodiode under reverse bias. a–f) Current mapping of a single Au nanoprism with a length of 100 nm smaller than that in Figure 2 (Scale bar, 50 nm). Note that a white dashed line is the upper edges of a Au nanoprism and a white solid line is the outermost Au nanoprism. These mappings were collected under an applied reverse bias of 0.8 *V*_tip_ under a,d) 250 mW cm^−2^, b,e) 500 mW cm^−2^, and c,f) 1000 mW cm^−2^ at 532 and 640 nm laser, respectively, exhibiting that the photocurrent at shorter wavelength (532 nm) increased significantly. g) Simulated absorbance spectrum of 100  and 136 nm long Au nanoprism is maximized at 541 nm and 670 nm, respectively. It revealed that the smaller Au nanoprism exhibits the shorter LSPR wavelength.

To get further insight into the amplification of the LSPR‐driven hot hole, we carried out a comparative experiment as a function of the size of the Au nanoprism. The AFM image in **Figure** **5** showed that photocurrents of a 100 nm long Au nanoprism, smaller than the Au in Figure 2, were directly observed at intensities of 250, 500, and 1000 mW cm^−2^ in incident laser wavelengths of 532 and 640 nm, respectively. Note that a white dashed line and a white solid line represent the trace of upper edges and the trace of the lower edge of a Au nanoprism, respectively. Dark current mapping under a reverse bias of 0.4 V reflected weak leakage current (Figure S5, Supporting Information). Photocurrent mappings under a reverse bias of 0.4 V at 532 and 640 nm light hardly increased compared to the dark currents. Under a reverse bias of 0.8 V, the photocurrents were more enhanced at the edge on LSPR excitation at a wavelength of 532 nm (Figure 5a–c) than 640 nm (Figure 5d–f). Concretely, the average photocurrent values, excluding dark currents at the edge, for the wavelengths of 532 and 640 nm under reverse biases of 0.4 and 0.8 V were 0.6, 6.4, 0.5, and 4.0 pA, respectively. Meanwhile, the average photocurrent values at the inner site under corresponding conditions exhibited 0.4, 3.0, 0.3, and 1.4 pA, respectively. The photocurrent difference between the inner site and the edge on 532 nm light was 1.2 and 1.1 times higher than the difference on 640 nm light at applied reverse biases of 0.4 and 0.8 V, respectively (Figure S6, Supporting Information). In contrast to the trend in Figure 2, hot hole flux of a 100 nm long Au nanoprism was enhanced under incident photons at a short wavelength at 532 nm. It is clear that the resonant wavelength of a 100 nm sized Au nanoprism is blueshifted compared to the 136 nm sized Au triangle. We attribute this to the shift of plasmonic resonance peaks as a function of the size of the plasmonic nanostructure. The theoretical absorbance in response to different sizes of Au nanoprisms by FDTD analysis accounts for the relationship between LSPR wavelength and the size of a single Au nanoprism (Figure 5g). The theoretical absorbance is in excellent agreement with the experimental results shown in Figure 5a–f. The calculated absorbance of 100 and 136 nm long Au nanoprisms is maximized at 541 and 670 nm, respectively. It is well established that the smaller plasmonic nanostructure exhibits a shorter LSPR wavelength.^[^ ^51^, ^52^ ^]^ Furthermore, we examined current‐profiles of the two different‐sized Au nanoprisms under various applied reverse bias and the LSPR excitation intensities (Figure S7, Supporting Information).

The difference in photocurrent that was observed between the interior of the Au nanoprism and the boundary region is associated with the increased transport distance. Now the transport distance of hot hole is directly related to the local thickness of Au. Although the thickness of Au at the interior of the Au nanoprism is close to that of the upper edge of the Au nanoprism, the photocurrent at the edge was enhanced in all directions. It indicates that LSPR‐field confinement spurred hot hole generation at the edge. Indeed, the fact that the photocurrent mapping exhibits the maximum at the upper edges (as shown in Figure S7, Supporting Information) implies that the enhanced electrostatic field at the edge of the Au nanoprism is mainly responsible for the amplification of hot holes.

Therefore, we can conclude that the LSPR‐coupled hot holes are responsible for the charge generation in the nanoarchitectures, inherited from a coherent coupling with LSPR‐field as a function of plasmonic nanostructured‐size. We consider that a controllable size‐dependent resonant coupling between surface plasmons and hot holes reflects the feasibility of effective hot carrier‐based photodetectors.

## 3 Conclusion

We have realized direct measurement of LSPR‐induced hot holes from triangular‐shaped Au nanostructures on p‐GaN support. By constituting enhanced hot hole‐driven photocurrents on a Au nanoprism/p‐GaN template under lesser energy photon excitation, we identified that the generation of plasmonic hot holes was successfully envisioned on the nanodiode. Furthermore, experimental and theoretical analysis jointly revealed that the spatial distribution of the |*E*|‐field of a single Au nanoprism intensified at the upper edge of the metallic Au nanostructure and induced the coupling between LSPR and field‐confinement, thus accelerating the generation of hot holes at the edge of the metallic nanomaterials. From the quantitative analysis for the photon‐to‐hot‐hole conversion efficiency, we confirmed that the edge surrounding a Au nanoprism acts as an erupting spot to increase LSPR‐driven hot holes. This suggests that the geometrical configuration of the plasmonic structure has the potential to extract them efficiently. Moreover, we demonstrated that amplification of hot holes at the edge was dependent upon the size of the Au nanoprism. This is attributed to the shift in the LSPR wavelength, thus suggesting the feasibility for photodiode application. We believe that this pioneering study will both provide insight into comprehension on the intrinsic relationship between hot holes and LSPR effect and serve as a starting point for the practical application of LSPR‐driven hot holes.

## 4 Experimental Section

##### Preparation of Polar C‐Plane GaN Templates

C‐plane (0002) p‐type GaN layers were grown on 2 inch c‐plane (0002) sapphire substrates using Aixtron G3 2600 metalorganic chemical vapor deposition (MOCVD). Trimethylgallium and ammonia (NH_3_) gases were used as precursor materials for Ga and N elements, respectively. Bicyclopentadienyl‐magnesium (Cp_2_Mg) was introduced as a p‐type dopant source. Prior to the growth of the p‐doped GaN films, the contaminants on the substrate were thermally cleaned at 1150 °C for 5 min under H_2_ ambient in the MOCVD reactor. Subsequently, 4 µm thick undoped GaN buffer layers were deposited at 1050 °C under N_2_ ambient, followed by the growth of 380 nm thick Mg‐doped p‐GaN films with a doping concentration of 5 × 10^18^ cm^−3^. The electrical activation of the templates was carried out at 750°C under N_2_ ambient for 1 min using rapid thermal annealing (RTA). Finally, Mg‐doped p‐type c‐plane GaN crystals were achieved.

##### Fabrication of Au Nanopattern on p‐GaN Support by Nanosphere Lithography

Au nanoprisms on p‐GaN were formed using nanosphere lithography with a polystyrene (PS) latex solution (Sigma‐Aldrich, Inc). The length of the nanoprism was adjusted by altering the size of the PS nanospheres. The monodisperse PS solution with a 10 wt% water was prepared by adding an equal amount of pure ethanol. Then, a slide glass was immersed slantwise into pure water in a vessel and 200 µL of 2% sodium dodecyl sulfate solution dropped onto the water surface. 15 µL of the PS solution was dropped onto the surface of the slide glass. The p‐GaN substrate was slowly immersed into the water of the vessel to take out a water‐drifting PS monolayer. After condensation of the PS monolayer, the PS monolayer was lifted off from the water surface and laid gently on the p‐GaN. After drying the p‐GaN substrate in air, 20 nm thick gold film on the substrate was deposited by e‐beam evaporator. The PS monolayer was removed by ultrasonic treatments, and Au nanoprisms on the p‐GaN formed in the hexagonal close‐packed array; the average length of one side of the nanoprism was 136 nm, corresponding to the size‐distribution histogram (see Figure S1, Supporting Information). For measuring hot holes as photocurrents, an ohmic‐contact on p‐GaN substrate was formed by heat treatment in air at 500 ˚C for 45 min after depositing 13 nm Ni/13 nm Au film on the p‐GaN to make a Ni/Au alloy.

##### Photoconductive AFM

Photoconductive AFM (pc‐AFM) was developed by modifying the sample stage with a fused silica prism and a lens to be a waveguide for back‐illuminating laser (OBIS series, Coherent). The incident laser entered perpendicular to the back of the sample. An external bias was applied to PtIr coated probe (PPP‐CONTPt, Nanosensors), then the photocurrent was collected by an electric circuit between the probe and an ohmic pad while the probe came into contact with the surface of a sample.

##### Numerical Analysis

A numerical analysis with FDTD method (FDTD Solutions, Lumerical) to confirm the |*E*|‐field distribution depending on the incident wavelengths was conducted. According to the average size of Au nanoprisms (136 ± 7 nm), the numerical simulations of theoretical |*E*|‐field distributions were performed with a 136 nm long Au nanoprism. Linearly polarized Gaussian field was used as a light source with the wavelength range from 400 to 850 nm. Further, from the simulations, the theoretical absorbance of a 100 and a 136 nm long Au nanoprism was obtained, respectively. The dielectric function of gold and the refractive indices of GaN substrate were obtained from the Johnson and Christy's parameters^[^ ^53^ ^]^ and a previous reference,^[^ ^54^ ^]^ respectively. A 0.5 nm mesh covered the Au nanoprism.

##### Statistical Analysis

For original research, the following information is provided: 1) The sample size was obtained from 45 Au nanoprisms in the SEM analysis, accompanied by an average side length of 136 ± 7 nm (please see Figure S1, Supporting Information). 2) All experimental absorbance of Au nanoprisms was measured after pre‐process subtracting the absorbance of the p‐GaN substrate.

## Conflict of Interest

The authors declare no conflict of interest.

## Supporting information

Supporting Information
