# Supplementary material for: In Situ Visualization of Localized Surface Plasmon Resonance‐Driven Hot Hole Flux
Source: Adv Sci (Weinh). 2020 Aug 6;7(20):2001148. doi: 10.1002/advs.202001148 (PMC7578898; doi:10.1002/advs.202001148)
Supplement: Supplementary file 1 — Supporting Information [file ADVS-7-2001148-s001.pdf]

© 2020 Wiley-VCH GmbH

ADVANCED SCIENCE
Open Access

Supporting Information

for *Adv. Sci.*, DOI: 10.1002/advs.202001148

In Situ Visualization of Localized Surface Plasmon Resonance-Driven Hot Hole Flux

*Hyunhwa Lee, Kyoungjae Song, Moonsang Lee,**
*and Jeong Young Park**

Supporting Information

In-situ visualization of localized surface plasmon resonance-driven hot hole flux

Hyunhwa Lee, Kyoungjae Song, Moonsang Lee, and Jeong Young Park**

H. Lee, K. Song, Prof. J. Y. Park

Department of Chemistry, Korea Advanced Institute of Science and Technology (KAIST), Daejeon 34133, Republic of Korea

Center for Nanomaterials and Chemical Reactions, Institute for Basic Science (IBS), Daejeon 31414, Republic of Korea

E-mail: jeongypark@kaist.ac.kr

Dr. M. Lee

Korea Basic Science Institute (KBSI), Daejeon 34133, Republic of Korea

E-mail: lms1015@kbsi.re.kr

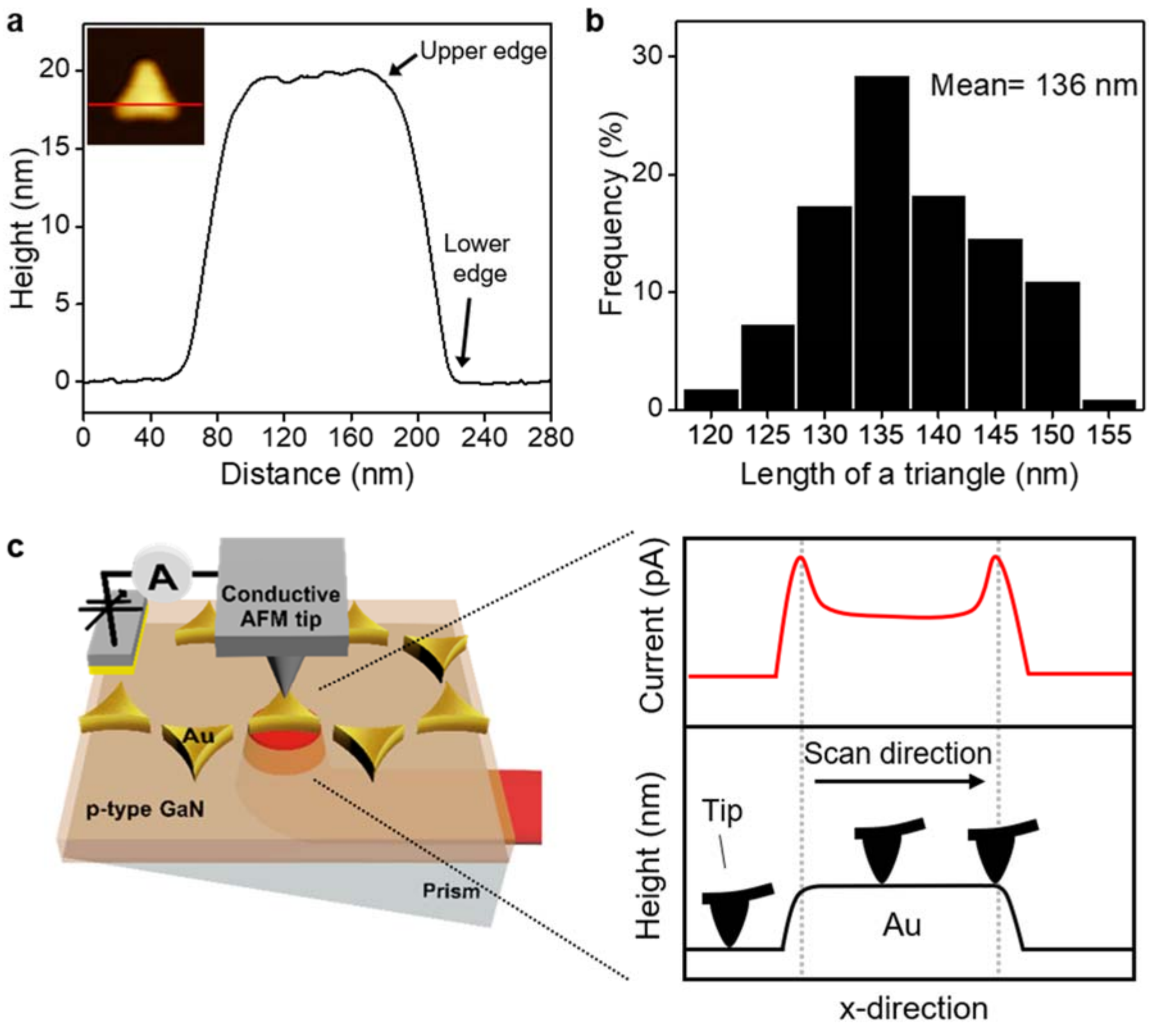

Supplemental Figure S1. Sample information. (a) Line profile of a single Au nanoprism with thickness of 20 nm. (b) Size-distribution histogram of Au nanoprisms, with an average side length of 136 ± 7 nm. (c) Schematic describing the process of detecting hot holes with pc-AFM. Once a hot hole is generated on a Au nanoprism, because of the short mean free path, these hot holes are transported through the Schottky barrier between Au and p-type GaN. The charge transport in Au/p-GaN platform occurs near the proximity of the AFM tip that gives rise to the local probing with nanoscale spatial resolution of the photocurrent mapping.

Thermionic emission equation

To verify the Schottky barrier height of the Au/p-GaN nanodiode, we fit the measured I–V curve to the thermionic emission equation.

The current density of the Au/p-GaN as a function of applied bias (V_a) is given by[1]

$$I = AA^{*}T^{2}\exp\left(-\frac{\Phi_b}{k_B T}\right)\left[exp\left(\frac{e_0(V_a - R_s I)}{\eta k_B T}\right) - 1\right]$$

where A is the active area, A* is the effective Richardson constant of p-GaN substrate, T is the temperature, Φ_b is the Schottky barrier height, η is the ideality factor, and R_s is the series resistance. The active area of the Au/p-GaN nanodiode is 8.4x10^{-11} cm^2 and the temperature is 300 K.

Supplemental Table S1. Fitting parameters in the thermionic emission equation.

Term	Value	Units
A*(p-GaN)	104 [ref. [2]]	A/cm^{2}K^2
T	295	K
K_B	8.62x10^{-5}	eV/K
KT/q	0.026	V
Area (A)	8.01x10^{-11}	cm^2
η	2.61	
Φ_b	1.03	eV
R_s	1.4	GΩ

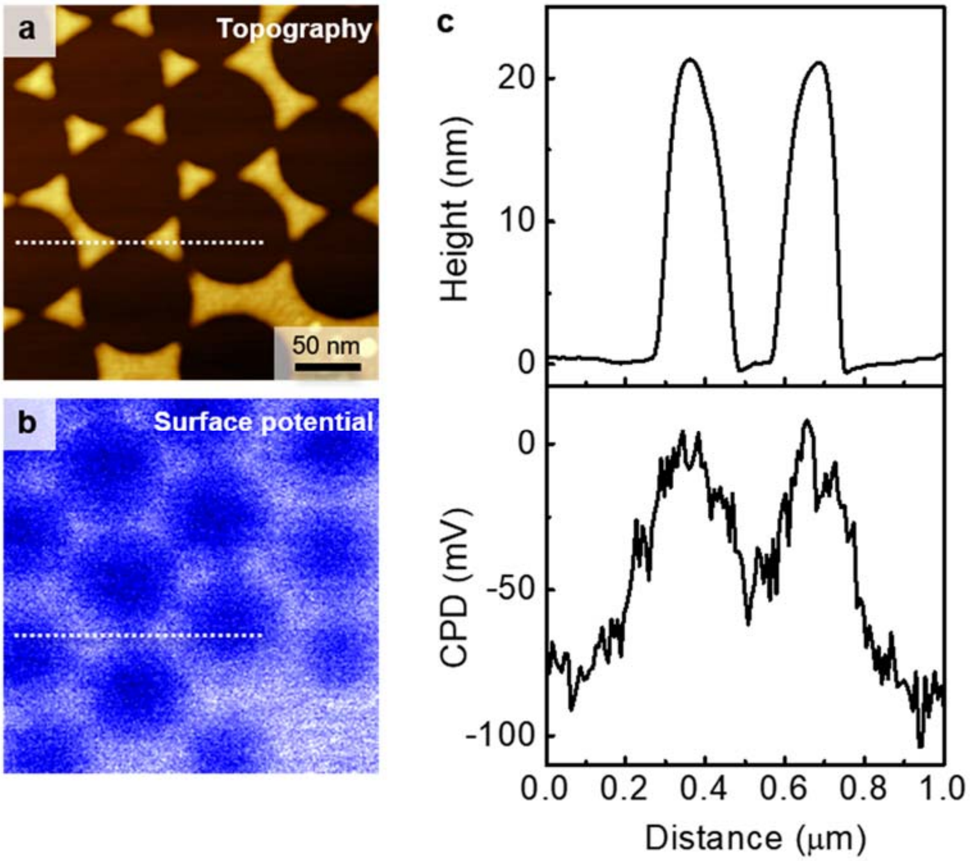

Supplemental Figure S2. KPFM measurement to verify Au/p-GaN Schottky junction. (a) The morphology of Au nanoprisms on p-GaN subatrate. (b) Surface potential image of Au nanoprisms and p-GaN substrate being measured by a PtIr coated tip. Note that bright surface-potential is obtained on Au nanopattern. The contact potential difference (V_{CPD}) between the tip and sample is defined as $V_{CPD} = (\Phi_{tip} - \Phi_{sample})/(-e)$, where Φ_{sample} and Φ_{tip} are the work functions of the sample and tip, and e is the electric charge[3]. The difference between Fermi energy levels of tip and sample generates an electrical force between tip and sample. (c) Topographic and CPD profile of white dashed line in (a) and (b) indicates the formation of a Schottky junction between Au and p-GaN substrate, because work function of the Au is lower than that of p-GaN.

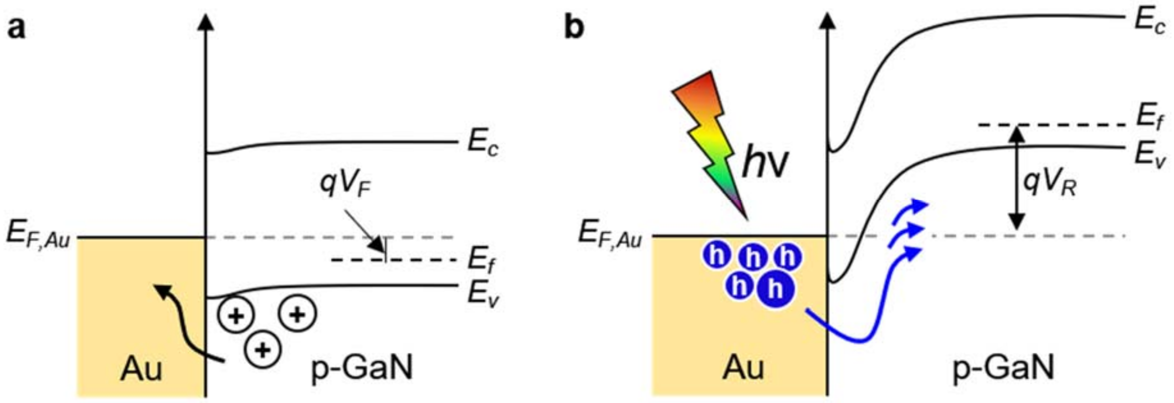

Supplemental Figure S3. Energy band diagram of Au/p-GaN Schottky junction under applied bias. (a) Schematic illustrating Schottky barrier formation between gold and p-GaN under forward bias, showing that the current is dominated by majority carrier transport from p-GaN to gold. (b) Energy band diagram under applying reverse bias shows that the Schottky barrier lowering effect increases the collection ratio of hot holes from gold to p-GaN under light illumination.

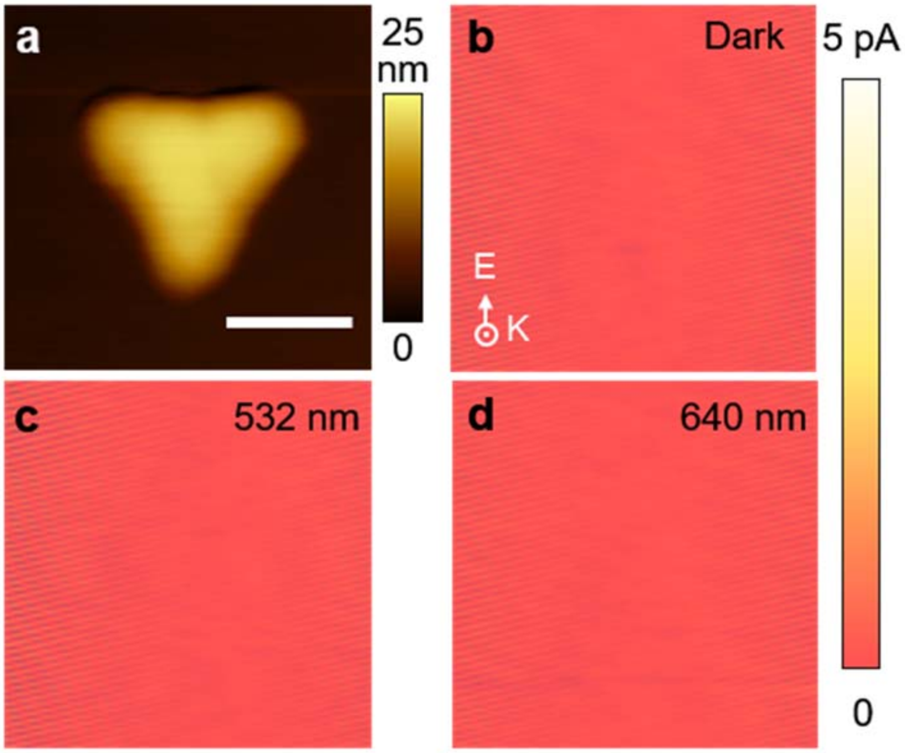

Supplemental Figure S4. Current mappings without applied bias. (a) Topography of a Au nanoprism on p-GaN in Fig. 2 (Scale bar, 100 nm). (b-d) Current mappings without applied bias corresponding to the topography in a, being measured in conditions of dark (b), 1000 mW/cm^2 at 532 nm (c) and at 640 nm illumination (d). In panels (c) and (d), hot holes as photocurrents are hardly captured because of their short mean free path.

Supplemental Table S2. Raw data of average currents on 136 nm long-Au nanoprism by pc-AFM experiment.

**Applied reverse bias: 0.5 V_{tip}**				
	Upper edge of Au		Interior of Au	
	Current (pA)	Photocurrent* (pA)	Current (pA)	Photocurrent* (pA)
dark	2.99	-	1.70	-
2.5 kW/m^2 at 532 nm	4.33	1.33	2.28	0.58
5.0 kW/m^2 at 532 nm	5.35	2.36	2.47	0.77
10 kW/m^2 at 532nm	6.67	3.68	2.84	1.14
2.5 kW/m^2 at 640 nm	5.38	2.39	3.15	1.45
5.0 kW/m^2 at 640 nm	6.50	3.50	3.36	1.66
10 kW/m^2 at 640 nm	8.42	5.42	3.98	2.28

**Applied reverse bias: 1.0 V_{tip}**				
	Upper edge of Au		Interior of Au	
	Current (pA)	Photocurrent* (pA)	Current (pA)	Photocurrent* (pA)
dark	45.59	-	14.04	-
2.5 kW/m^2 at 532 nm	53.92	8.33	15.27	1.23
5.0 kW/m^2 at 532 nm	57.04	11.45	16.49	2.45
10 kW/m^2 at 532nm	59.43	13.84	17.65	3.61
2.5 kW/m^2 at 640 nm	63.70	18.11	23.01	8.97
5.0 kW/m^2 at 640 nm	67.44	21.85	25.70	11.66
10 kW/m^2 at 640 nm	73.25	27.66	27.30	13.26

Photocurrent*: The average photocurrent values excluding dark currents.

From the table, we describe the role of enhancement factors: (1) Applied bias (0.5 V_{tip} and 1.0 V_{tip}) lowers the Schottky barrier height at the interface of Au/p-GaN, which creates an environment where hot holes can be easily detected. Also, we can verify that hot hole flux is increased by the increment of the reverse bias beyond the dark currents. (2) LSPR-field near 640 nm (on resonance) increases the light absorption of Au nanoprisms more than at 532 nm (off resonance), consequently resulting in enhanced hot hole flux.

Calibration of the tip-sample contact area

The contact area between the conductive AFM tip and the sample was calculated as follows.

The contact area A was expressed as[4]

$$A = \pi \left(\frac{R}{K} \cdot (L + 2\pi R\gamma) \right)^{2/3}$$

where K is the combined elastic modulus of tip and sample.

$$\frac{1}{K} = \frac{3}{4}\left[\frac{1 - \nu_t^2}{E_t} + \frac{1 - \nu_s^2}{E_s} \right]$$

where R is the curvature of tip (25 nm) and L is effective normal load (0.02 nN). $2\pi R\gamma$ is the adhesion force between tip and sample, and γ is the work of adhesion. E_t and E_s are Young's modulus, and ν_t and ν_s are the Poisson ratios of the tip and sample, respectively. By using the elastic constants and the adhesion force of material, the contact area is calculated depending on the position of tip (Table S3). The contact area at the upper edge of Au nanoprism is 1.08 times larger than that on the inner Au.

Supplemental Table S3. Elastic constants of the materials and calculated contact area.

**Material**	**Poisson's ratio**	**Young's modulus (GPa)**	**Adhesion force (nN)**	**Contact area (nm^2)**
PtIr (tip)	0.37	233	-	-
p-GaN	0.23	295	10.60	3.88
Inner Au	0.40	79	5.52	4.08
Edge Au	0.40	79	6.22	4.43

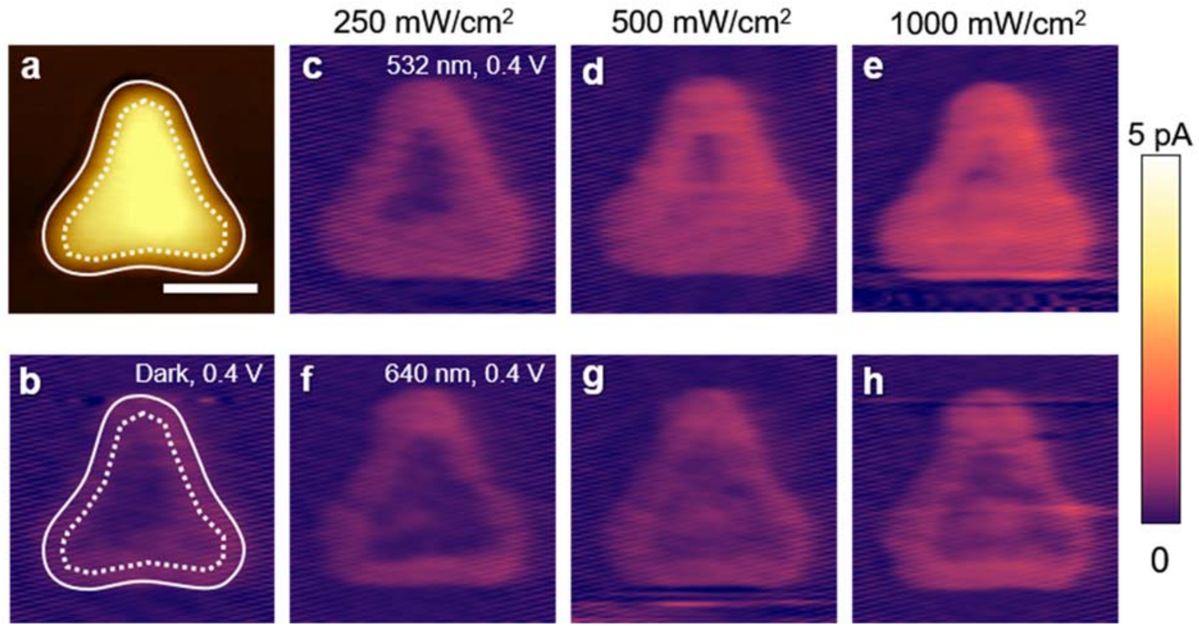

Supplementary Figure S5. Photocurrent mapping of a small Au nanoprism under reverse bias of 0.4 V_{tip}. (a) Topography of single Au nanoprism with a length of 100 nm in Fig. 5 (Scale bar, 50 nm). Here, a white dashed line and a white solid line represent the trace of upper edges, and that of the lower edge of a Au nanoprism, respectively. (b-h) Current mapping collected by an applied reverse bias of 0.4 V_{tip} in the dark (b), under 250 mW/cm^2 (c,f), 500 mW/cm^2 (d,g) and 1000 mW/cm^2 (e,h) at 532 nm and 640 nm laser, respectively. It shows that the photocurrent at the shorter wavelength (532 nm) increased significantly because of the LSPR effect in Fig. 5g.

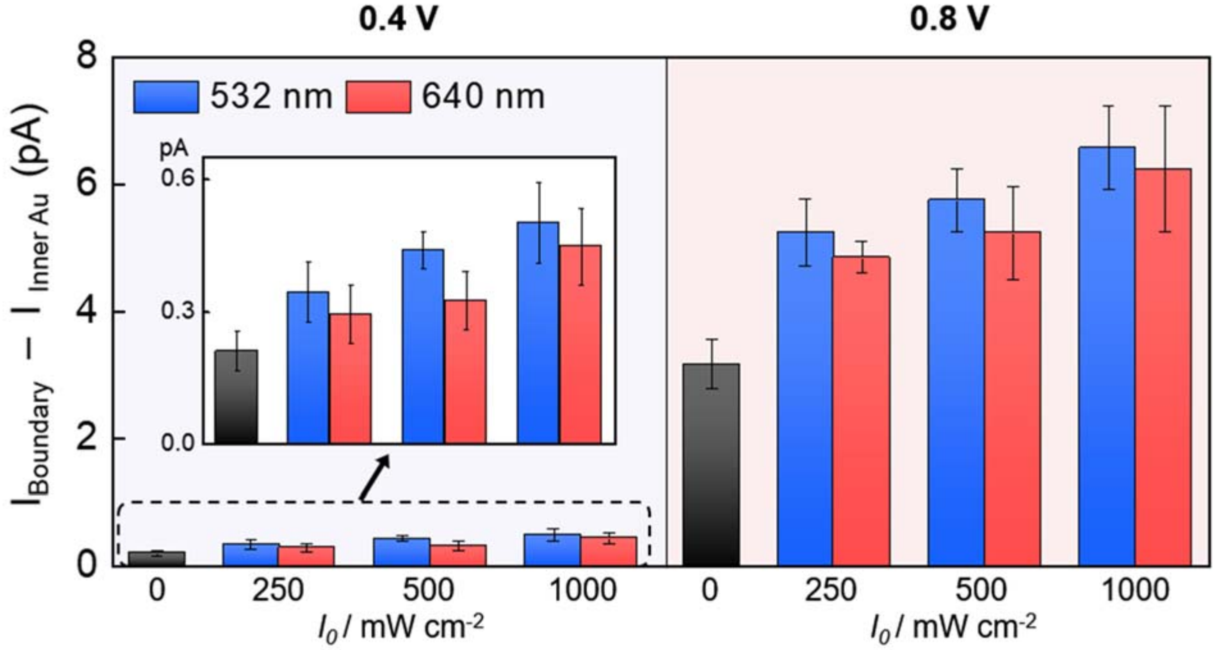

Supplemental Figure S6. Enhanced photocurrents at the upper edge of 100 nm-sized Au nanoprism under reverse bias of 0.4 V_{tip} and 0.8 V_{tip}. Photocurrents at the edge under the incident light at 532 nm in Fig. 5 are more amplified than those at 640 nm, because the LSPR peak of the Au nanoprism was shifted to short wavelengths by the small size of the Au nanoprism.

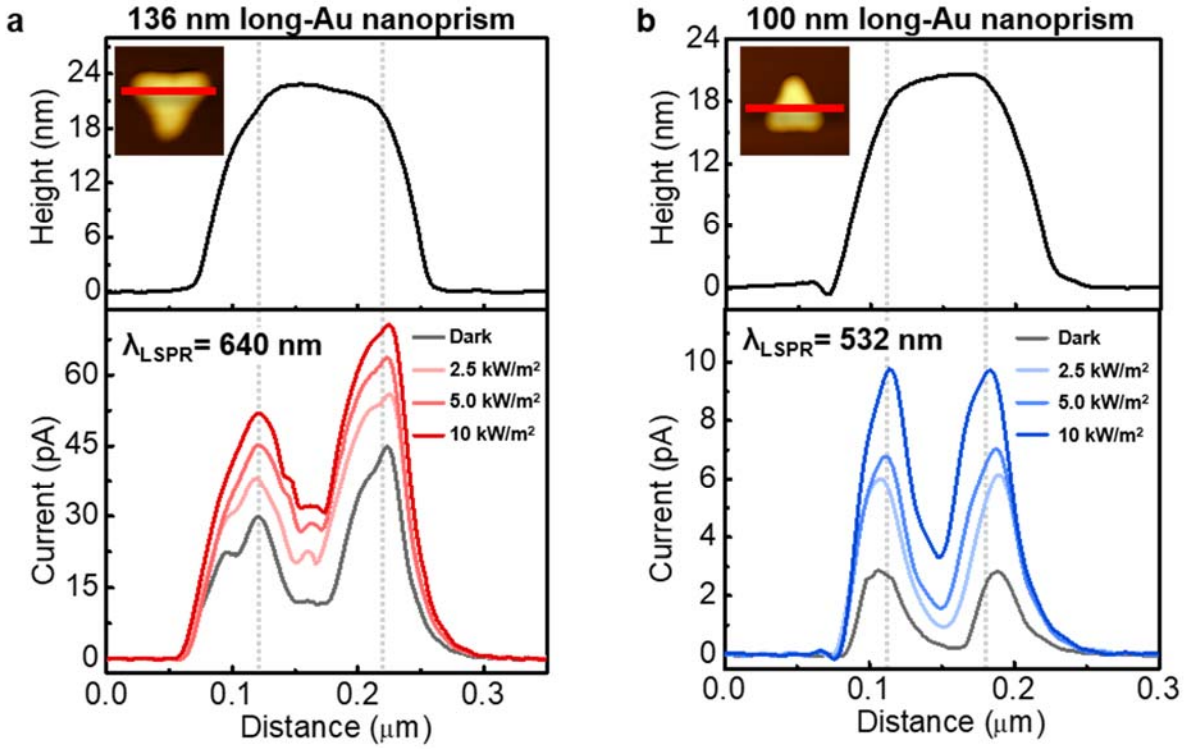

Supplemental Figure S7. Surface plasmon-driven hot hole enhancement at the upper edge beyond the increased contact area and field effect. (a) The height and current profile of a 136 nm long Au nanoprism on p-GaN under reverse bias of 1.0 V_{tip}. It shows field-enhancement at the edge depending on the increased intensities of LSPR excitation at 640 nm. Likewise, (b) current profile of 100 nm long-Au nanoprisms on p-GaN under reverse bias of 0.8 V_{tip} corresponds to field-enhancement at the edge under LSPR excitation at 532 nm. These profiles in (a) and (b) suggest that hot hole flux is amplified at the upper edge beyond the increased tip-sample contact area and field effect in the dark.

References

[1] K.K.N. S.M. Sze, *Physics of Semiconductor Devices*, John Wiley & Sons, Place **2006**.
[2] Y.-J. Lin, *Appl. Phys. Lett.* **2005**, 86, 122109.
[3] W. Melitz, J. Shen, A.C. Kummel, S. Lee, *Surf. Sci. Rep.* **2011**, 66, 1.
[4] R.W. Carpick, D.F. Ogletree, M. Salmeron, *J. Colloid Interface Sci.* **1999**, 211, 395.
